# Endoscopic surgery versus conservative treatment for the moderate-volume hematoma in spontaneous basal ganglia hemorrhage (ECMOH): study protocol for a randomized controlled trial

**DOI:** 10.1186/1471-2377-12-34

**Published:** 2012-06-07

**Authors:** Xin Zan, Hao Li, Wenke Liu, Yuan Fang, Junpeng Ma, Zhigang Lan, Xi Li, Xin Liu, Chao You

**Affiliations:** 1 Department of Neurosurgery, West China Hospital, Sichuan University, No.37 Guoxue Alley, Chengdu, Sichuan Province, People's Republic of China, Zip code: 610041

**Keywords:** Endoscopic surgery, Conservative treatment, Moderate-volume hematoma, Spontaneous basal ganglia hemorrhage

## Abstract

**Background:**

Spontaneous intracerebral hemorrhage is a disease with high morbidity, high disability rate, high mortality, and high economic burden. Whether patients can benefit from surgical evacuation of hematomas is still controversial, especially for those with moderate-volume hematomas in the basal ganglia. This study is designed to compare the efficacy of endoscopic surgery and conservative treatment for the moderate-volume hematoma in spontaneous basal ganglia hemorrhage.

**Methods:**

Patients meet the criteria will be randomized into the endoscopic surgery group (endoscopic surgery for hematoma evacuation and the best medical treatment) or the conservative treatment group (the best medical treatment). Patients will be followed up at 1, 3, and 6 months after initial treatment. The primary outcomes include the Extended Glasgow Outcome Scale and the Modified Rankin Scale. The secondary outcomes consist of the National Institutes of Health Stroke Scale and the mortality. The Barthel Index(BI) will also be evaluated. The sample size is 100 patients.

**Discussion:**

The ECMOH trial is a randomized controlled trial designed to evaluate if endoscopic surgery is better than conservative treatment for patients with moderate-volume hematomas in the basal ganglia.

**Trial registration:**

Chinese Clinical Trial Registry: ChiCTR-TRC-11001614

(http://www.chictr.org/en/proj/show.aspx?proj=1618)

## Background

Spontaneous intracerebral hemorrhage (SICH) is a disease with high morbidity, high disability rate, high mortality, and high economic burden [1,2]. Its annual incidence is about 20 cases per 100,000 population [3,4]. More than 70% patients end up with death or dependence [5,6].

The main clinical management of SICH includes surgical and medical treatment. Whether patients can benefit from surgery is still controversial, especially for those with basal ganglia hemorrhage, which is the major subtype of SICH [7]. Surgical evacuation of hematomas can relieve the mass effect and reduce the harmful substance released during their coagulation, liquefaction, and resolution [8-10], but surgical procedures may also injure the brain tissue, and cause other operative complications. These disadvantages reduce the benefit of surgery. Some clinical trials were carried out to compare surgery with medical treatment. The most famous trial is the STICH trial conducted by Mendelow et al. [5]. 1033 patients were randomized. After 6 months follow-up, 26% of patients in the early surgery group had a favourable outcome compared with 24% in the group of initial conservative treatment. Early surgery did not show significant advantages. However, in this study less than 25% of the operations were minimally invasive. Trials of stereotactic surgery or craniopuncture were also carried out [11-13]. But none of the trials and systematic reviews provide sufficient evidence for the choice of treatments [6,14-16].

In clinical practice, neurologists and neurosurgeons tend to choose conservative treatment for patients with small-volume hematomas, and surgery for those with large-volume hematomas [17]. However, when the volume of the hematoma exceeds 60 ml, neither of the methods give much help [18]. Treatments for moderate-volume hematomas have the most uncertainty. Some recently randomized trials indicate these patients may benefit from minimally invasive surgery [13,19]. Kim et al. [19] randomized 387 patients with basal ganglia or thalamus hematomas to stereotactic-guided evacuation (group A) or conservative treatment (group B), and the mean hematoma volume was 23.1 ml. At the end of 6 months follow-up, the mean score of modified Barthel index was 90.9 in group A and 62.4 in group B, and the mean score of modified Rankin scale was 1.2 in group A and 3.0 in group B. Patients received stereotactic-guided evacuation had better clinical outcomes and motor function.

Endoscopic surgery is one of the minimally invasive ways for hematoma evacuation. Compared with other surgical methods, it has advantages as follows. A). The hematoma evacuation is performed under direct vision of neurosurgeons. It’s much easier to spot any bleeding site, so that the risk of postoperative rebleeding is lower. B). The evacuation rate of hematomas is higher because of a wider vision. C). This also leads to less brain retraction and brain tissue injury. D). The operation time is shorter. Cho et al. [20] carried out a randomized controlled trial which compared endoscopic surgery, stereotactic aspiration and craniotomy. The results showed endoscopic surgery had the highest hematoma evacuation rate, the lowest mortality and complication rate, and shorter waiting time than stereotactic aspiration. However, there are few randomized controlled trials which contrast endoscopic surgery and conservative treatment. The trial completed by Auer et al. [21] in 1989, showed 28% lower of the mortality in the endoscopic surgery group than the medical treatment group after 6 months follow-up. But this trial did not restrict the volume of hematomas.

It's not clear whether endoscopic surgery gives this type of patients better outcomes, so we have designed this study to find more clues for the clinical management of SICH.

## Methods

### Study objective

To compare the efficacy of endoscopic surgery and conservative treatment for the moderate-volume hematoma in spontaneous basal ganglia hemorrhage.

### Inclusion criteria

· Spontaneous basal ganglia hemorrhage confirmed by computed tomography(CT).

· The volume of the hematoma is between 20 ml to 40 ml. Small intraventricular hemorrhage is not counted.

· Admission within 36 hours from the ictus.

· Age between 15 to 75 years old.

· Glasgow Coma Score(GCS) ≥ 8.

### Exclusion criteria

· Any sign of brain herniation, including deep coma, unilateral or bilateral pupil dilation, abnormal posture, or unstable vital signs.

· Hemorrhage caused by aneurysm, vascular malformations, tumor apoplexy, trauma, or cerebral infarction. Computed tomography angiography will be performed.

· Large intraventricular hematomas which occupy half of the cross section area of the lateral ventricle on CT scans, or hydrocephalus caused by hematocele.

· Primary intraventricular hemorrhage.

· Dysfunction of blood coagulation, thrombocytopenia, or history of taking any kind of drugs which affect coagulation function in the past 40 days.

· Before the ictus, the patient has severe physical or mental illness which causes significant disability, or the patient has other severe comorbidity.

· The patient is pregnant.

### Sample size

The rate of the favourable outcome of patients with basal ganglia hemorrhage is about 30% for conservative treatment [5,6] and 60% for endoscopic surgery [22-24]. A sample size of 86 will be required with a significance level of 5% (2-sided) and a power of 80%. In consideration of the loss to follow-up, the sample size is enlarged to 100.

### Randomization

The minimization method is adopted for the patients allocation, and it’s performed by a minimization software. When the qualification of a patient is confirmed, the investigator sends the basic information of the patient to a special trialist who is responsible for patients randomization. After the information of the patient is imported, the software will randomize him/her into the endoscopic surgery group or the conservative treatment group. The presetted variables for minimization are age, hematoma volume, and admission GCS.

The allocation of patients cannot be changed. If the patients' condition deteriorates during conservative treatment, and the surgical indications are clear, hematoma evacuation will be performed after fully informed consent.

### Blinding

Both patients and surgeons cannot be blinded. In order to minimize the measurement bias, the following procedures are made. A). Patient outcomes will be measured by two special investigators who are not involved in the allocation and the treatment. Before the outcome measurement starts, patients, their relatives, and the related medial staff will be told not to reveal any information about the treatment. B). Every patient will have a gauze covered on the hemorrhage side of the head to conceal the scar or baldness. C). Because the follow-up forms and the treatment forms are printed on the same set of Case Report Form (CRF), these two investigators must hand in a copy of the original score sheets to the Quality Monitoring Board (QMB), before they are allowed to get the CRF of the patient. D). The statistical analysis will be made by a statistician who doesn't participate in the implementation of the trial, and the treatment information of the groups will be blinded.

### Measurement of the hematoma volume

The ABCs method is adopted to measure the hematoma volume: Volume(ml) = (A × B × C)/2[25]. “A” is the long axis of the hematoma on the CT slice where the hematoma has the largest area. “B” is the longest axis of the hematoma perpendicular to the long axis on the same CT slice. “C” is the product of the interslice distance and the count of slices on which the hematoma is visible. The intraventricular hematoma is not counted. The rebleeding or hematoma expansion is defined as a 10 ml increase between two CT scans.

### Treatments

#### *Endoscopic surgery group*

This group of patients will receive endoscopic surgery and the best medical treatment. The surgery will be performed as soon as possible after the randomization, and the medical treatment will be started immediately. Neurosurgeons will decide surgical approaches according to the location, size, and shape of hematomas. The hematomas will be evacuated as much as possible with the help of the angled endoscope, but the stiffly attached clot won’t be removed by force in order to prevent unnecessary damage, and the restricted intracavity operation will be the principle during the hematoma evacuation.

A “Rebleeding test” will be performed after the hemostasis. For patients with hypertension, the blood pressure will be slowly elevated to the level before the anesthesia, and for other patients, a raise of 20 ~ 30 mmHg will be gained. The anesthetist will maintain this level of blood pressure for 10 minutes. Only if there is no new bleeding site, neurosurgeons can start to close.

#### *Conservative treatment group*

The best medical treatment will be provided to this group of patients right after the admission. A “multistep blood pressure control” will be carried out. The range of the first reduction is limited to 20% of the admission level for patients with hypertension, and it should be accomplished in half an hour. If the patients' systolic blood pressure exceeds 225 mmHg, the goal is lower than 180 mmHg. The level of blood pressure will be maintained for 8 hours and gradually reach the normal standard in 72 hours.

After patients are stabilized, the rehabilitation will be started as early as possible.

### Follow-up

Patients will be followed up at 1, 3, and 6 months after initial treatment.

### Outcome assessment

Both investigators have been trained at least 20 hours by multimedia, bedside practice, and a qualification exam, before they start to evaluate patient outcomes.

#### *Primary outcomes*

The primary outcomes of the study are the Extended Glasgow Outcome Scale (GOSE)[26] and the Modified Rankin Scale (mRS) at 6 months after initial treatment. Both scales will be measured with structured interviews.

#### *Secondary outcomes*

The secondary outcomes include the National Institutes of Health Stroke Scale(NIHSS) and the mortality after 6 months since initial treatment.

#### *Other outcome*

The Barthel Index (BI) will also be evaluated.

### Ethics

This study is conducted in accordance with the Declaration of Helsinki and guideline for Good Clinical Practice. All patients and their relatives are fully informed about the trial, and they have had a copy of the informed consent form. The trial is approved by the Biological and Medical Ethics Committee (BMEC) of West China Hospital (2011Reviewed-No.92).

### Data collection and management

The allocation data of patients is kept by the trialist who specializes in randomization. Neurosurgeons should fill in CRF except the follow-up forms, which must be completed by another two investigators trained for outcome assessment. Double input will be used in the data entry. The QMB and the principal investigator will check and examine the data, then the database will be locked and sent to a statistician.

### Adverse and severe adverse events

Adverse event (AE) is any undesirable incident happening to patients during the study. It will be recorded on CRF, including a description of the event, the correlation with the trial, the starting date, the ending date, actions taken and their efficacy, and the patient outcome. Severe adverse event (SAE) is defined as the event of death or vegetative state. Severe disability is not included, because basal ganglia hemorrhage tends to cause severe disability. SAE will also be recorded on CRF. All AEs and SAEs will be reported to the QMB and the BMEC, and the time limit for SAE report is 24 hours.

### Statistical analysis

Both Intention To Treat (ITT) and Per-Protocol (PP) analysis will be performed. The level of significance is 0.05, and Power is 80%. The quantitative data will be analyzed by *t*-test. Chi-square test will be used to analyze the categorical data. For the ordinal data, Wilcoxon rank test is adopted. And survival analysis will be carried out for further comparison of mortality. Logistic regression will be used to adjust the effect of multivariables.

## Discussion

Spontaneous basal ganglia hemorrhage is very likely to cause death, independence, disability, and lead to a great financial burden, so it’s always a hot issue in neurology and neurosurgery. There are already more than 10 randomized controlled trials referring to the treatment choices of SICH, but we still designed this study due to the following reasons. A). To evacuate or not, it’s controversial, especially for moderate-volume hematomas. B). It’s reasonable to evacuate the hematoma considering its mechanical, chemical, and biological harm to the brain tissue [8-10]. C). As a minimally invasive technique, endoscopic surgery may reveal the benefit of the hematoma evacuation, which is probably covered by the secondary injury caused by traditional craniotomy. D). There is a lack of randomized controlled trial focusing on endoscopic surgery for the moderate-volume hematoma in spontaneous basal ganglia hemorrhage.

For patients in the conservative treatment group, if their condition deteriorates because of hematoma expansion or rebleeding during the trial, and the surgical indications are clear, then hematoma evacuation should be performed. The crossover will be analyzed in both ways: ITT and PP.

Surgical approaches for the hematoma evacuation in the basal ganglia include the temporal approach, the frontal approach, and the transsylvian approach. The last one is often performed under the microscope, so it won’t be applied in this study. The temporal approach usually provides the shortest distance from the cortex to the hematoma, but it may cause injury to the visual pathway, and if the hematoma is elliptical, neurosurgeons may fail to evacuate the frontal part of the hematoma because of the blind angle. In the frontal approach, the trajectory of endoscope is parallel to the long axis of the hematoma, so neurosurgeons get a better view through this approach, and it’s advised to be used for hematomas larger than 50 ml [27]. However, for small and moderate volume hematomas, much further distance is needed to reach them by the frontal approach, which means more brain tissue damage. Also, the aspect ratio of moderate-volume hematomas is more approximate to 1:1 than the large ones, which makes fewer blind angle through the temporal approach. In this study, surgical approaches will be decided according to the location, size, and shape of hematomas.

In conclusion, the ECMOH trial is a randomized controlled trial designed to assess if endoscopic surgery is better than conservative treatment for patients with moderate-volume hematomas in the basal ganglia.

## Abbreviations

ECMOH: Endoscopic surgery versus conservative treatment for the moderate-volume hematoma; SICH: Spontaneous Intracerebral Hemorrhage; CT: Computed Tomography; GCS: Glasgow Coma Score; CRF: Case Report Form; QMB: Quality Monitoring Board; GOSE: Extended Glasgow Outcome Scale; mRS: Modified Rankin Scale; NIHSS: National Institutes of Health Stroke Scale; BI: Barthel Index; BMEC: Biological and Medical Ethics Committee; AE: Adverse Event; SAE: Severe Adverse Event; ITT: Intention To Treat; PP: Per-Protocol.

## Competing interests

The authors declare that they have no competing interests.

## Authors’ contributions

CY is the principle investigator, has initiated the trial, been part of the trial design and protocol writing. XZ has been part of the trial design and drafted the protocol. HL, WL, YF, XL, JM, ZL, and XL have been part of the trial design and the protocol writing. All authors have read, edited, and approved the final manuscript.

## Pre-publication history

The pre-publication history for this paper can be accessed here:

http://www.biomedcentral.com/1471-2377/12/34/prepub
